# Effect of hydrolyzed red worm ( *Eisenia foétida*) on production parameters in red tilapia ( *Oreochromis sp.*)

**DOI:** 10.12688/f1000research.154622.1

**Published:** 2025-01-15

**Authors:** Fabian Gerardo Muñoz García, Nelson Vivas Quila, Luis Fernando Londoño Franco, Crispulo Perea Roman, José Luis Hoyos Concha

**Affiliations:** 1 Zoot, Universidad del Cauca, Popayán, Cauca, Colombia; 2 Zoot, Universidad del Cauca, Popayán, Cauca, Colombia; 3 Colombian Polytechnic Jaime Isaza Cadavid, Medellín, Antioquia, Colombia; 4 Universidad del Cauca, Popayán, Cauca, Colombia; 5 Universidad del Cauca, Popayán, Cauca, Colombia

**Keywords:** Energy, hydrolyzed, fish, production, protein

## Abstract

**Background:**

Conventional fish feed based on fish meal, meat, and soy cake presents procurement difficulties and high costs, affecting the profitability and sustainability of the aquaculture industry.

**Objective:**

To evaluate the effect of hydrolyzed red worm (HRW- *Eisenia foétida*) in red tilapia ( *Oreochromis sp.*) diet on production parameters.

**Methods:**

The study was conducted at the aquaculture farm of the Politécnico Colombiano Jaime Isaza Cadavid (PCJIC) at 780 m.a.s.l.,with an average temperature of 28 °C. Ninety red tilapia fingerlings, averaging 7,5±0,5 g, were distributed in nine aquariums containing 75 liters of water. Fish underwentweight and size measurements at the beginning and end of the trial. They were fed experimental diets to apparent satiation three times daily. Water quality parameters and productive rates of growth and nutrient utilization were measured. The experimental design included three treatments with three replicates each: T1 (control diet, 0% hydrolysate inclusion), T2 (10% hydrolysate inclusion), and T3 (20% hydrolysate inclusion). ANOVA (p<0,05) was applied to growth and nutrient utilization variables, with mean comparisons using α<0,05 in SPSS version 25.

**Results:**

Significant differences (p<0,04) were found between the control diet T1 (0% inclusion) and T2 (10% inclusion) in favor of weight gain (31,87 g). There were no statistical differences in size increase (p<0,217). As HRWinclusion increased, feed consumption decreased, likely due to higher hydrolyzed protein availability. Feed conversion rates showed significant differences (p<0,001) between T2 and T3 compared to T1, indicating better assimilation of the hydrolyzed protein. T2 and T3 also showed better protein and energy efficiency (p<0,001), demonstrating the hydrolyzed protein’s nutritional quality and assimilation. Diet cost decreased with higher hydrolyzed inclusion (p<0,034).

**Conclusion:**

Inclusion 10% and 20% hydrolyzed red worms significantly improved production parameters and reduced costs, making it a viable alternative for feeding red tilapia for small and medium-scale producers.

## Introduction

Currently, the scarcity of raw materials and high costs for fish feeding necessitate the use of new or innovative resources to address this issue, ^1^ which should also be environmentally friendly and sustainable. In this regard, vermiculture emerges as an alternative in bioconversion processes, such as Hydrolyzed Red Worms (HRW), for inclusion in fish diets. ^2^ Additionally, it is known that the red worm ( *Eisenia foétida*) is an important source of nutrients: proteins (62%), fiber (7%), fats (8%), ash (9%), and energy 3.9 kcal. ^3^ Likewise, the hydrolyzed contains free amino acids and low molecular weight peptides, promoting absorption and making them highly digestible due to their nutritional quality. ^4^ Consequently, this study aims to obtain an alternative food source by utilizing the nutrients from the red worm to determine a nutritional and productive effect on the development of the early stages of red tilapia ( *Oreochromis sp.*) *.*

## Methods

**Location**: The study was conducted at the experimental and aquaculture production farm of Politécnico Colombiano Jaime Isaza Cadavid - PCJIC, located in the municipality of San Jerónimo-Antioquia-Colombia, at coordinates 6° 26′ 49.88″ North Latitude and -75° 43′ 55.42″ West Longitude, at an altitude of 780 meters above sea level, with an average temperature of 28°C and relative humidity of 50%, classified as Tropical Dry Forest (TDF).

### Experimental biological material

**Animals:** Red tilapia fingerlings ( *Oreochromis sp.*) with an average body mass of 7.5±0.5 g from the PCJIC farm. Additionally, 8 kg of HRW ( *Eisenia foétida*) is provided by the Institución Educativa Noroccidente Popayán farm.

**Hydrolyzed preparation:** Two kilograms of worms without bedding residues were mixed with 2.5% formic acid (85% m/v) to lower the pH and facilitate the hydrolytic action of endogenous enzymes. The mixture was supplemented with 0.25% sodium benzoate as an antimicrobial and 0.1% butylhydroxytoluene as an antioxidant. Three replicates were performed. ^2, 5^ The hydrolyzed was placed in sealed plastic containers with a 30% headspace and left at room temperature for 10 days (enzymatic hydrolysis) until the product was ready for subsequent use. ^1, 6, 7^

### Experimental environment

Ninety red tilapia fingerlings were weighed and randomly distributed into nine glass aquariums measuring 36 × 35 × 80 cm, with a capacity of 75 liters. The aquariums were pre-disinfected with 50 ppm sodium hypochlorite and a 100 ppm iodine solution on the ceiling, walls, nets, and floors. There were placed 10 fingerlings per aquarium. The setup was as follows: three aquariums for diet 1 (control with 0% hydrolyzed worm), three for diet 2 (10% hydrolyzed worm), and three for diet 3 (20% hydrolyzed worm). Each treatment was triplicate, with an average body mass of 7.5 ± 0.5 g per fingerling.

The aquariums were equipped with an aeration system consisting of a diffuser stone connected by a hose to an air pump, model-51, 2,5 HP (Sweetwater brand).

The water volume in the aquariums was supplied by the Guaracú stream, which feeds the entire fish station and meets the conditions suitable for fish survival. The water was pre-aerated and filtered using a Hydrofiltro, Mardal brand, model FCM 100, 2 HP (USA). The light-dark photoperiod cycle was 12:12. The water temperature in the aquariums ranged from 24.5 to 27.5 °C, with an average of 26 ± 0.0 °C. The physicochemical parameters of the water were monitored daily with a multiparameter device, Hanna brand, model HI98194, ^8^ ensuring they met the requirements for the species. The water temperature was kept constant according to the farm environment.

### Experimental feeding

An acclimatization period of 10 days was conducted, during which the experimental diets were supplied to adapt the digestive system to the type of experimental food. During the study phase, the evaluation diets were provided for apparent satiation three times daily (8:00 a.m., 12:00 p.m., and 4:00 p.m.). The food was offered according to the biomass of the animals, considering an average water temperature of 26 °C and voluntary food ingestion as determined by the following equation 1. ^9^

Constant temperature at 26 ° CFood ingestion(gfishday)=0.15∗weight(g)^0.600 (1)

Uneaten food and feces were removed daily in the morning (9:00 a.m.) and in the evening (5:00 p.m.) by siphoning, and 60% of water changes were performed in the aquariums. ^1^

### Experimental diets

Analysis of hydrolyzed red worm ( *Eisenia foétida*) was conducted at the Universidad Nacional de Medellín to determine the nutrient content: dry matter (AOAC 934.01; AOAC, 1990), crude protein by Kjeldahl method (AOAC, 1987), ether extract (EE) by gravimetry (AOCS, 1998; AOAC, 1990), calcium (AOCS, 1998; AOAC 985.35; AOAC, 2005), phosphorus (AOAC 995.11; 2012), and the gross energy (GE) was determined precisely using the CAL2k ^®^ bomb calorimeter system, in the institution’s research laboratory. ^10^ The analysis results are presented in Table 1.

**Table 1.** Proximate analysis of hydrolyzed red worm ( *Eisenia foétida*).

Variable	Quantity g/100 g
Dry matter digestibility	82±0.6
Crude protein	59±0.2
Ether extract	7±0.6
Digestible energy	4.817±0.0 Cal
Phosphorus	0.8±0.2
Calcium	0.4±0.06

Source: Current study.

The dry matter digestibility values obtained for the Hydrolyzed Red Worm (HRW) were greater than 82%. This can be attributed to a higher degree of liquefaction, resulting in simple peptides, dipeptides, and tripeptides from the hydrolysis process, thereby increasing the amount of dry matter available for fish feed (see Table 1).

It is important to highlight that the crude protein content was above 59%, due to the quality of the animal-derived raw material and probably the acidic conditions promoting greater lysis of strong peptide bonds like glycine and alanine. These amino acids have properties that ensure soluble protein stability under ideal temperature conditions. ^11^ Similar results were reported by Muñoz et al., ^6^ in their study on the preparation and characterization of hydrolyzed red worm ( *Eisenia foétida*), where they demonstrated the nutritional quality of the hydrolyzed and its protein potential for use in animal feed.

The composition in grams of the ingredients used in the experimental diets is presented in Table 2. Additionally, the inclusion levels of the hydrolyzed red worm are shown, with three replicates conducted for each treatment. ^2, 12^

**Table 2.** Composition in grams of diets with red worm hydrolyzed red worm ( *Eisenia foétida*).

Diet ingredients	T1 0%	T2 10%	T3 20%
Fish meal	35±0.00	23±0.00	26.5±0.00
Wheat flour	3±0.00	3±0.00	3.9±0.00
Vegetable oil	3±0.00	3±0.00	3±0.00
Dicalcium phosphate	0.4±0.01	0.4±0.00	0.89±0.00
DL-methionine	0.2±0.01	0.2±0.00	0.69±0.00
Premix1	1±0.00	1±0.00	1±0.00
Tryptophan	0.3±0.00	0.3±0.00	0.28±0.0
Corn bran	9±0.00	9±0.00	7±0.00
Soy cake	25±0.00	26.3±0.0	10.5±0.00
Yellow corn flour	5±0.00	6±0.00	9±0.00
Wheat muffin	13.1±0.00	11.1±0.00	10.24±0.00
Cassava flour	3±0.00	3±0.00	4.5±0.00
Bentonite	1±0.00	1.7±0.00	1.5±0.00
Salt	1±0.00	2±0.00	1±0.00
Hydrolyzed red worm (HRW2)	0±0.00	10±0.00	20±0.00
Total	100±0.0	100±0.0	100±0.0

Source: Current study.

^1^ Premix=premix of vitamins, minerals, and additives.

^2^ HRW= Hydrolyzed red worm.

The formulation of the experimental diets was carried out in the Rheology Laboratory of the Universidad del Cauca. The following ingredients were used: hydrolyzed red worm ( *Eisenia foétida*), fish meal, soy cake, yellow corn flour, wheat flour, corn bran, cassava flour, wheat muffin, vegetable oil, premix (vitamins, minerals, and additives), among others. Bentonite was used as a binder. Additionally, three isoproteic diets, T1, T2, and T3, were prepared for the evaluation, with a protein level of 36% ( Table 3) and isoenergetic with a digestible energy of 3,25 cal/g. These protein-energy levels are recommended for red tilapia ( *Oreochromis sp.*). ^13, 14^

**Table 3.** Composition of diets with red worm hydrolyzed ( *Eisenia foétida*).

Nutrient	T1 0%	T2 10%	T3 20%
**Crude Protein**	36.7±0.0	36.6±0.0	36.6±0.0
**Ether extract**	7.5±0.0	7.3±0.0	8.0±0.0
**Gross fiber**	2±0.0	2±0.0	1.6±0.0
**Ash**	14.9±0.0	11.3±0.0	11.1±0.0
**Digestible energy (Kcal/100g)1**	325.5±0.0	325.5±0.0	325.6±0.0
**Calcium**	2.1±0.0	1.5±0.0	1.9±0.0
**Phosphorus**	1.2±0.0	1±0.0	1.2±0.0

Source: Current study.

^1^ Kcal/100 g = Kilocalories per 100 grams.

The diets were prepared by incorporating the dry ingredients, followed by the addition of hydrolyzed, vegetable oil, and water. The mixtures were then homogenized in a Kitchen Aid mixer with a capacity of 10 kg, packed in polypropylene bags, and stored for 24 hours at 10°C to equilibrate the moisture. ^15^ The diets were then processed in a Haake Polylab OS twin-screw extruder. The pellets underwent a drying process in a Centricol Ltda equipment, series 0827, at 50°C for 4 hours until reaching 9% moisture. Variables such as compression, durability, water stability, floatability, and water absorption rate were monitored. ^16^

The resulting pellets were 2 mm in length and 1.3 mm in diameter. ^15^ Subsequently, the physical and mechanical characteristics of the pellets were evaluated. ^13, 16^

### Feeding protocol

The fish were fed to apparent satiation three times daily (8:00 a.m., 12:00 p.m., and 4:00 p.m.) for 30 consecutive days. Daily records of the feed supplied in each treatment were maintained to determine the feed consumption during the evaluation period. ^17, 18^

### Diet cost

The cost of the diet was determined based on the value of each ingredient used in the formulation for each treatment (see Table 4). Additionally, the processing cost of the feed was considered to quantify the value of each experimental diet in Colombian pesos, which was then converted to US dollars. ^12, 13^ Table 4 shows each ingredient in kilograms and the corresponding value for each treatment. Furthermore, the total value in pesos and dollars (currency exchange in 2022) is indicated.

**Table 4.** Value of ingredients in diets with red worm hydrolyzed ( *Eisenia foétida*).

Raw materials	Cost kg RM ^1^	T1 0%		T2 10%		T3 20%	
		Qty/kg ^2^	Value	Qty/kg	Value	Qty/kg	Value
**Fish meal**	3.250	0.35	1.137.50	0.23	747.50	0.265	861.25
**Wheat flour**	850	0.03	25.50	0.03	25.50	0.039	33.15
**Vegetable oil**	3.600	0.03	108.00	0.03	108.00	0.03	108.00
**Dicalcium phosphate**	2.800	0.004	11.20	0.004	11.20	0.00894	25.03
**DL-methionine **	14.000	0.002	28.00	0.002	28.00	0.0069	96.60
**Premix**	20.000	0.01	200.00	0.01	200.00	0.01	200.00
**Tryptophan**	156.000	0.003	468.00	0.003	468.00	0.0028	436.80
**Corn bran**	1.000	0.09	90.00	0.09	90.00	0.07	70.00
**Soy cake**	2.062	0.25	515.50	0.263	542.31	0.105	216.51
**Yellow corn flour**	1.350	0.05	67.50	0.06	81.00	0.09	121.50
**Wheat muffin**	437	0.131	57.25	0.111	48.51	0.10236	44.73
**Cassava flour**	1.200	0.03	36.00	0.03	36.00	0.045	54.00
**Bentonite**	2.380	0.01	23.80	0.017	40.46	0.015	35.70
**Salt**	1.000	0.01	10.00	0.02	20.00	0.01	10.00
**HRW**	1.000	0.00	0.00	0.1	100.00	0.2	200.00
**Maquila**			$356.00		$356.00		$356.00
**Diet cost “pesos”**		$3.134.25		$2.902.47		$2.869.27	
**USD diet cost per year (2022)**		US$ 0.84		US$ 0.78		US$ 0.77	

Source: Current study.

^1^ RM= Row Material.

^2^ Qty/kg= quantity per kilogram.

Two samplings were conducted, one at the beginning and one at the end of the trial, where the weight of the animals was obtained to evaluate the performance of each treatment. The evaluated variables are presented in detail in Table 5.

**Table 5.** Variables evaluated in *Oreochromis sp.,* fed with red worm hydrolyzed ( *Eisenia foétida*).

Variable	Rates
Growth	Weight gain
	Size increase
	Thermal growth coefficient
Nutrient utilization	Feed consumption
	Feed conversion
	Protein efficiency rate
	Energy efficiency rate

Source: Current study.

### Growth rates

The evaluated growth rates were: weight gain (WG) (Eq. 2), size increase (SI) (Eq. 3), and thermal growth coefficient ratio (TGC) (Eq. 4), as estimated according to Refs. 12, 19. Biometries were performed at the beginning and end of the study. The obtained data were independently compiled into tables to standardize the observation and analysis of the growth and nutrient utilization parameters. ^2, 20^

Weight gain=WG(g)=final weight–initial weight (2)

Size increase=SI(cm)=final size–initial size (3)

Thermal growth coefficient = TGC

$$\text{TGC}=\frac{100*\left(\text{final weight}\;1/3–\text{initial weight}\;1/3\right)\;}{\mathrm{Sum}\;\text{of}\;\mathrm{day}\;\text{temperature in °C}}$$ (4)

Nutrient Utilization Variables: these variables were estimated according to equations 5, 6 and 7. ^21– 23^

Feed Conversion Rate (FCR)

Feed Conversion Rate=FCR=total feed ingestion(g)/weight gain(g) (5)

Protein Efficiency Rate (PER)

Protein Efficiency Rate:PER(g)=weight gain/protein consumed (6)

Energy Efficiency Ratio (EER)

Energy Efficiency Ratio:EER(kcal/g)=weight gain/energy consumed (7)

### Statistical analysis

A randomized block design (RBD) was applied, evaluating 3 treatments with three replicates each (each aquarium with 10 fish) considered as an experimental unit to determine growth parameters and nutrient utilization. Each variable was reviewed through the application of an analysis of variance (p<0.05). Additionally, Duncan’s test was used for mean comparison with α=0.05 as a significant statistical difference, using SPSS version 23 to statistically compare the results of each treatment during the study. ^2, 24^

## Results and Discussion

It is important to mention that during the research, no mortality was observed and the monitored physicochemical parameters of the water were within the suitable limits for red tilapia ( *Oreochromis sp.*) production. ^13, 25^ The mean values of dissolved oxygen were (5.55 ± 0.10 mg L ^−1^), pH (7.25 ± 0.17), carbonate alkalinity (0.50 ± 0.12 mmol L ^−1^), chemical oxygen demand (5.60 ± 0.42 mg L ^−1^), biological oxygen demand (3.98 ± 0.10 mg L ^−1^), ammonium (0.08 ± 0.22 mg L ^−1^), nitrites (0.10 ± 0.00 mg L ^−1^), and nitrates (0.07 ± 0.18 mg L ^−1^), indicating that the inclusion of the hydrolyzed did not affect fish mortality or the productive variables. ^1, 12^

Table 6 presents the evaluated variables of growth rate and nutrient utilization in red tilapia with the calculated variance statistics and Duncan’s test.

**Table 6.** Growth and use of nutrients in *Oreochromis sp.*, fed with hydrolyzed *Eisenia foétida.*

Variable	T1	T2	T3	ANOVA P< 0.05
Growth				
WG ^1^ (g)	81.13±13.55 ^b^	113±9.68 ^a^	90.5±16.49 ^ab^	0.047
SI ^2^ (cm)	0.3567±0.2743 ^a^	0.8430±0.1305 ^a^	0.60±0.4187 ^a^	0.217
TGC ^3^ (%)	1.71±0.11 ^a^	2.2±0.06 ^a^	1.94±0.36 ^a^	0.830
Nutrient utilization				
FCR ^4^ (g)	1.47±0.04 ^a^	1.14±0.04 ^b^	1.15±0.06 ^b^	0.001
PER ^5^ (g)	1.89±0.06 ^b^	2.43±0.08 ^a^	2.42±0.11 ^a^	0.001
EER ^6^ (kcal/g)	2.14±0.06 ^b^	2.75±0.09 ^a^	2.74±0.13 ^a^	0.001
Diet cost (US $ kg ^−1^) ^7^	0.86 ± 0.00 ^a^	0.82 ± 0.00 ^b^	0.81 ± 0.00 ^b^	0.034

Source: Current study. Different letters in the same row indicate statistical differences (p<0.05).

^1^ WG= Weight Gain.

^2^ SI= Size Increase.

^3^ TGC= Thermal Growth Coefficient.

^4^ FCR= Feed Conversion Rate.

^5^ PER= Protein Efficiency Rate.

^6^ EER= Energy Efficiency Ratio.

^7^ (US $ kg ^−1^) = Dollars per kilogram.

Different letters in the same row indicate statistical differences (p<0.05).

**Figure 1.**
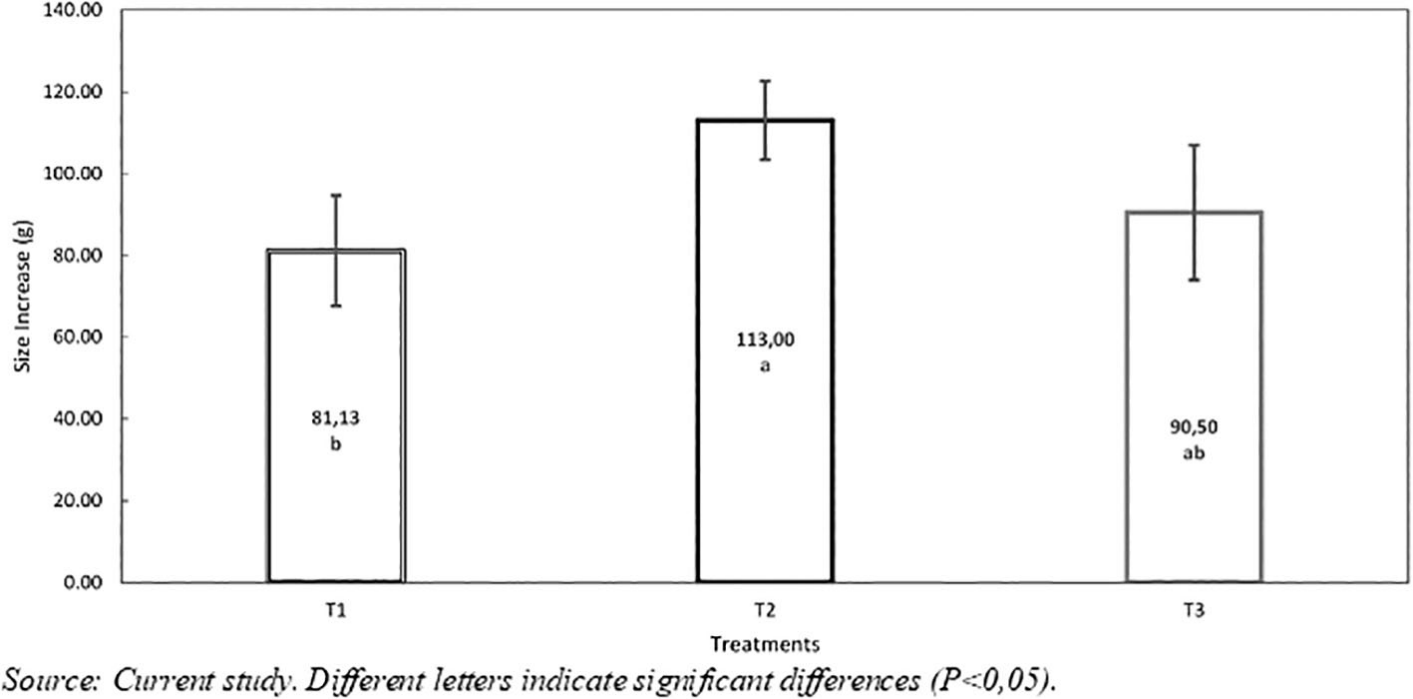
Weight gain of red tilapia ( *Oreochromis* sp.) fed with hydrolyzed red worm ( *Eisenia foétida*).

***Weight Gain*:** According to the variance analysis shown in Table 6, the weight gain in treatments T2 (10% inclusion) and T3 (20% inclusion) did not show significant differences between them. However, there was a statistical difference (p<0.047) between the control diet (T1: 81.13±13.55) and Treatment T2 (113±9.68) (see Figure 1). In summary, T2 presented a weight gain of 31.87 g compared to the control diet (T1). Similar results to this study were reported by Perea et al., ^19^who evaluated the inclusion of 0, 10, 20, and 30% fish waste silage in the feeding of *Oreochromis sp.*, obtaining better results for the 10% and 20% inclusions, with weight gains of 80.75 and 89.94 g respectively.

Similarly, Yucra, ^20^ in his study on feeding trout ( *Oncorhynchus mykiss*) with biological silage from trout viscera, obtained similar weight gain results of 37.36 g and 35.74 g for treatments T2 (27.9% silage) and T1 (commercial), respectively, demonstrating the nutritional quality of the silage. Furthermore, silage can replace fish meal by up to 27.9%, standing out as a low-cost alternative.

**Figure 2.**
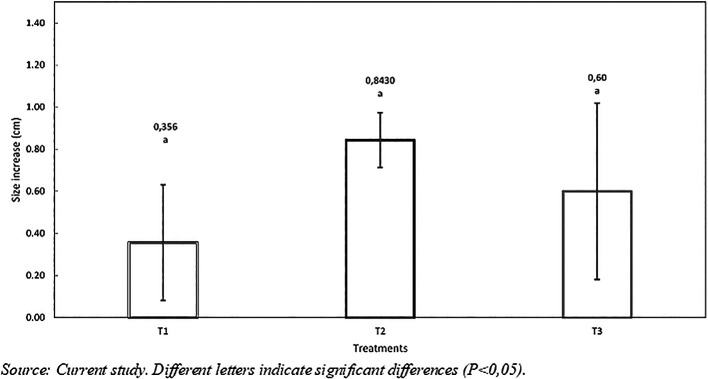
Size increasse of red tilapia ( *Oreochromis* sp.) fed with hydrolyzed red worm ( *Eisenia foétida*).

*Size increase*: The ANOVA analysis in Table 6 shows no statistical differences (p<0.217) between treatments. However, the best result for size increase was obtained with treatment T2, which presented a greater growth of 0.486 cm and 0.243 cm compared to T1 and T3, respectively (see Figure 2).

Consequently, increasing the levels of hydrolyzed red worm inclusion in the diets of red tilapia fingerling improved weight gain and to a lesser extent, growth (size). This can be attributed to factors favoring weight and growth gain, including protein quality, feeding rate, and appropriate water temperature (26 °C). ^26^ Furthermore, the decrease in growth between T2 and T3 can be attributed to the excess protein in the diet with 20% hydrolyzed inclusion, likely due to an extra energetic effect, leading to lower consumption and progressive growth reduction as the extra ingested energy is used for other purposes rather than weight gain and size increase. ^23^

Similarly, research by Perea et al., ^27^ on juvenile tilapia sp. with the inclusion of fish silage (10, 20, and 30%, respectively), reported that fingerlings fed with diets containing higher silage inclusion showed greater weight gain and size increase, possibly due to the quantity and quality of amino acids present in the silage, making it a viable alternative in animal feeding.

*Thermal Growth Coefficient*: Table 6 shows no statistical differences in the thermal growth coefficient between treatments in the study. However, the best values were obtained with treatments T2 (10% inclusion) and T3 (20% inclusion) compared to T1 (0% inclusion). The thermal growth coefficient is an index that relates the effect of water temperature on the productive performance of fish. ^2^ In the present study, the temperature (26 ± 0.00 °C) was maintained within the adequate range for red tilapia ( *Oreochromis sp.*) production and was similar for each evaluated treatment. ^19^ Therefore, the differences in the thermal growth coefficients are directly attributed to the effect of the diets.

Moreover, the study by Mora et al., ^21^ which evaluated the effect of crude protein levels (28, 32, and 36%) in extruded commercial feeds on the growth of *Leiarius marmoratus* (catfish) fingerlings, demonstrated no significant differences in the thermal growth coefficient (TGC), with values ranging between 0,667 and 0,712. This contrasts with the values of the present study, which supports the quality of the hydrolyzed used in this study.

Another study by Aguilar et al, ^28^ which examined the effect of feed processing (extruded versus pelleted) on the productive performance including the thermal growth coefficient in *Oreochromis niloticus* (nile tilapia) fingerlings and juveniles, found similar results to our study and reported no significant differences in this parameter during the feeding phases, with values of 0.206±0.0025 and 0.203±0.0031 in the fingerling stage, and 0.103±0.0028 and 0.098±0.0021 in the juvenile stage. These values are lower than those obtained in diets with hydrolyzed red worm ( *Eisenia foétida*) inclusion, highlighting the hydrolyzed’s significant energy and nutritional content for use in animal feeding.

The following results relate to different productive parameters of nutrient utilization rates: feed conversion rate, protein efficiency rate, and energy efficiency ratio with the inclusion of hydrolyzed red worms in the feeding of red tilapia ( *Oreochromis sp.*).

Evaluation of Nutrient Utilization Rate: Table 6 presents the nutrient utilization evaluation, which determined indices such as feed conversion rate (FCR), protein efficiency rate, energy efficiency ratio, and diet cost ratio.

**Figure 3.**
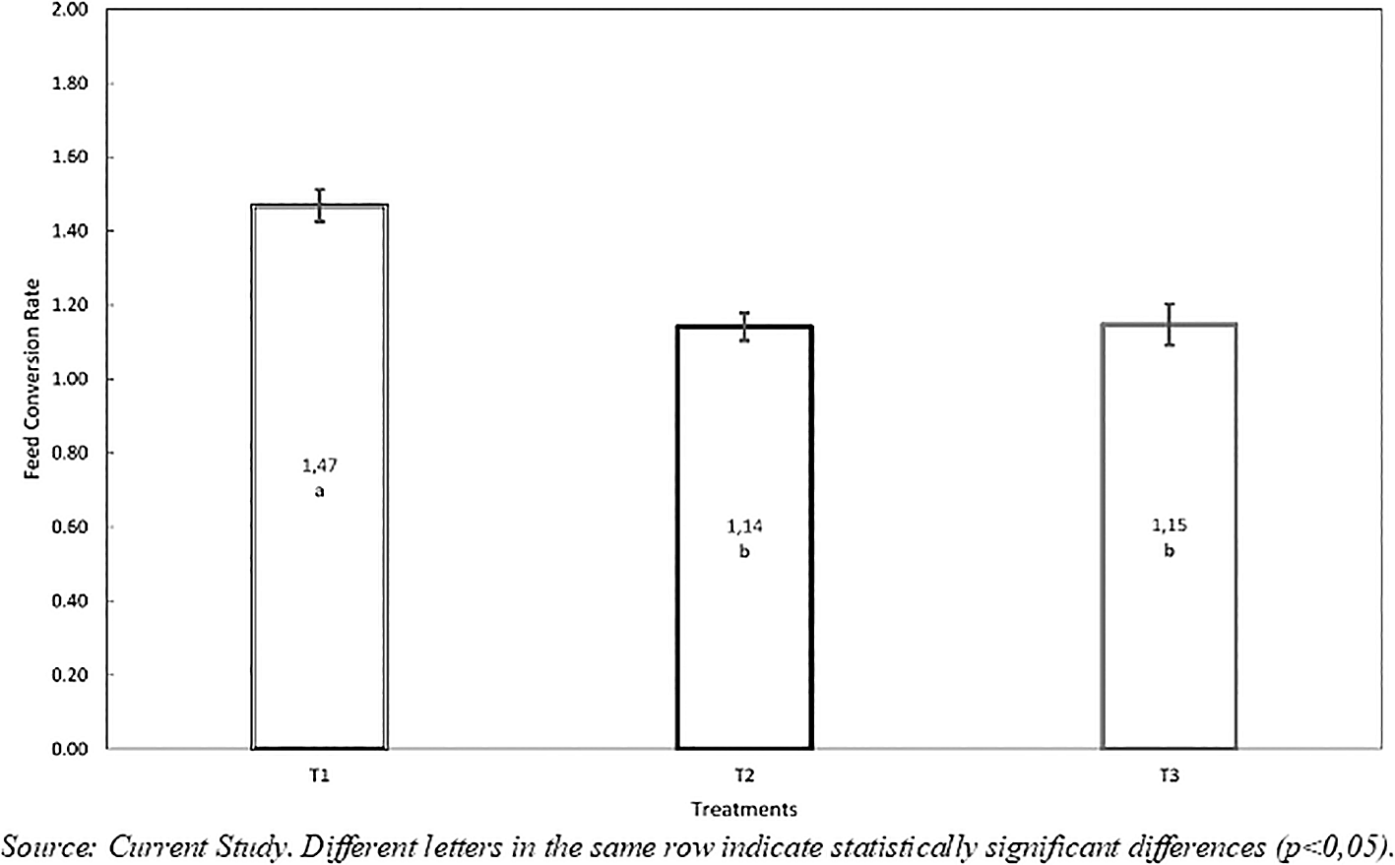
Feed conversión rate with inclusión of hydrolyzed red worm ( *Eisenia foétida*).

*Feed Conversion Rate*: As shown in Table 6, the variance analysis (p<0.001) demonstrated significant differences between treatments. Additionally, Duncan’s mean test determined significant differences (p<0.05) between the diet without hydrolyzed inclusion (T1) and the diets containing hydrolyzed T2 (10%) and T3 (20%), with values of 1.47±0.04, 1.14±0.04, and 1.15±0.06, respectively (see Table 6 and Figure 3). However, treatments T2 and T3 were similar and did not show statistical differences between them.

It is worth noting that in feed conversion, lower values are better from a productive standpoint. ^29^ The trend in this variable indicates that an inclusion above 10% hydrolyzed significantly improves feed conversion. The best feed conversion rates achieved with 10% and 20% inclusion levels are likely due to the nutritional quality of the hydrolyzed (protein, energy, and amino-lipid profile), which enhances the metabolism of red tilapia ( Figure 3). This confirms the advantages of including the hydrolyzed in fish diets. ^1, 2^ It also supports the acceptance and palatability of the feed by the fish.

*Protein Efficiency Rate (PER)*

**Figure 4.**
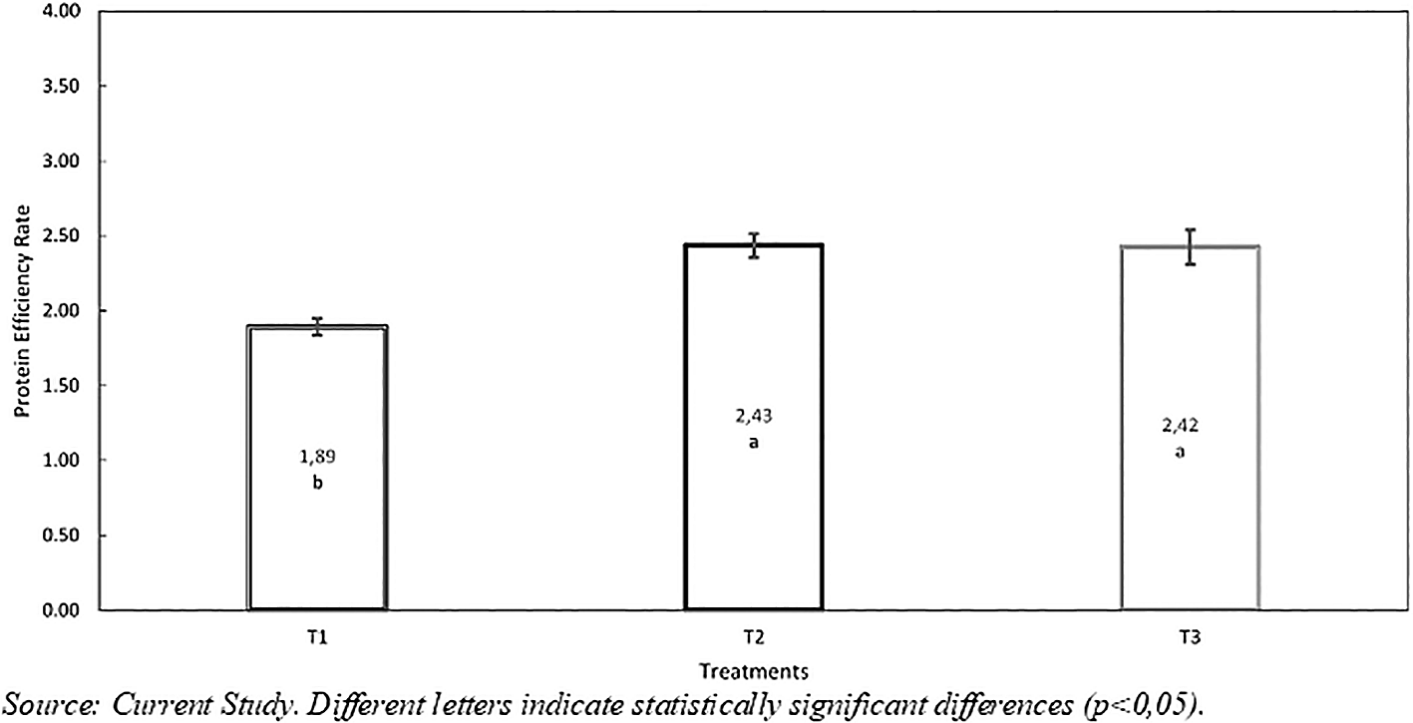
Protein efficiency rate with inclusión of hydrolyzed red worm ( *Eisenia foétida*).

According to Table 6, there were statistical differences (p<0,001) between the treatment T1 without hydrolyzed inclusion (HRW) compared to T2 and T3. T2 (2,43%) showed better protein efficiency, followed by T3 (2,42%) and T1 (1,89%). Furthermore, T2 and T3 demonstrated better performance by 0,53 and 0,54 grams compared to T1, respectively ( Figure 4), favoring improved protein digestion and metabolism, likely due to the quantity and nutritional quality of the hydrolyzed. ^1, 6^ This demonstrates that appropriate inclusion levels can constitute an important protein source in red tilapia production, a fish that seems to efficiently utilize the nutrients present in the hydrolyzed.

The results obtained are possibly due to the greater availability of hydrolyzed energy and protein, resulting from the acidification process that facilitates enzymatic hydrolysis, increasing low molecular weight proteins, releasing amino acids, and raising unsaturated fatty acid content, thus optimizing nutrient digestibility and utilization. ^12, 23^ The higher quantity and quality of nutrients in the fish’s intestinal lumen improve metabolism and absorption, enhancing feed conversion. ^12^

The protein efficiency rate results obtained in this study contrast with those cited in a study on *Clarias gariepinus* (African catfish) by Llanes et al., ^30^ where they evaluated the partial replacement of fishmeal with meat silage with inclusion levels of 10% and 20%, obtaining values of 2.17 and 1.50, respectively. Additionally, they differ and are lower than those reported by Martinez-Castillo et al., ^31^ who evaluated the zootechnical performance of *Piaractus brachypomus* (cachama) fingerlings fed diets with different gross energy levels between 3750 and 4440 kcal/kg, obtaining results of 3.97 and 4.80. Similarly, they differ from those presented by Llanes y Parisi., ^32^ who evaluated hydrolyzed fishery products in *Clarias gariepinus* (African catfish), reporting values of 3.1 and 3.2 in protein efficiency. Furthermore, they contrast with the results obtained by Murillo et al., ^33^ who reported in their study on growth, efficiency, and composition of *Oreochromis aureus* fed with red worm ( *Eisenia foétida*) that there was no significant difference in weight gain or length. However, there was a significant difference in feed conversion (p<0.05).

*Energy Efficiency Ratio*

**Figure 5.**
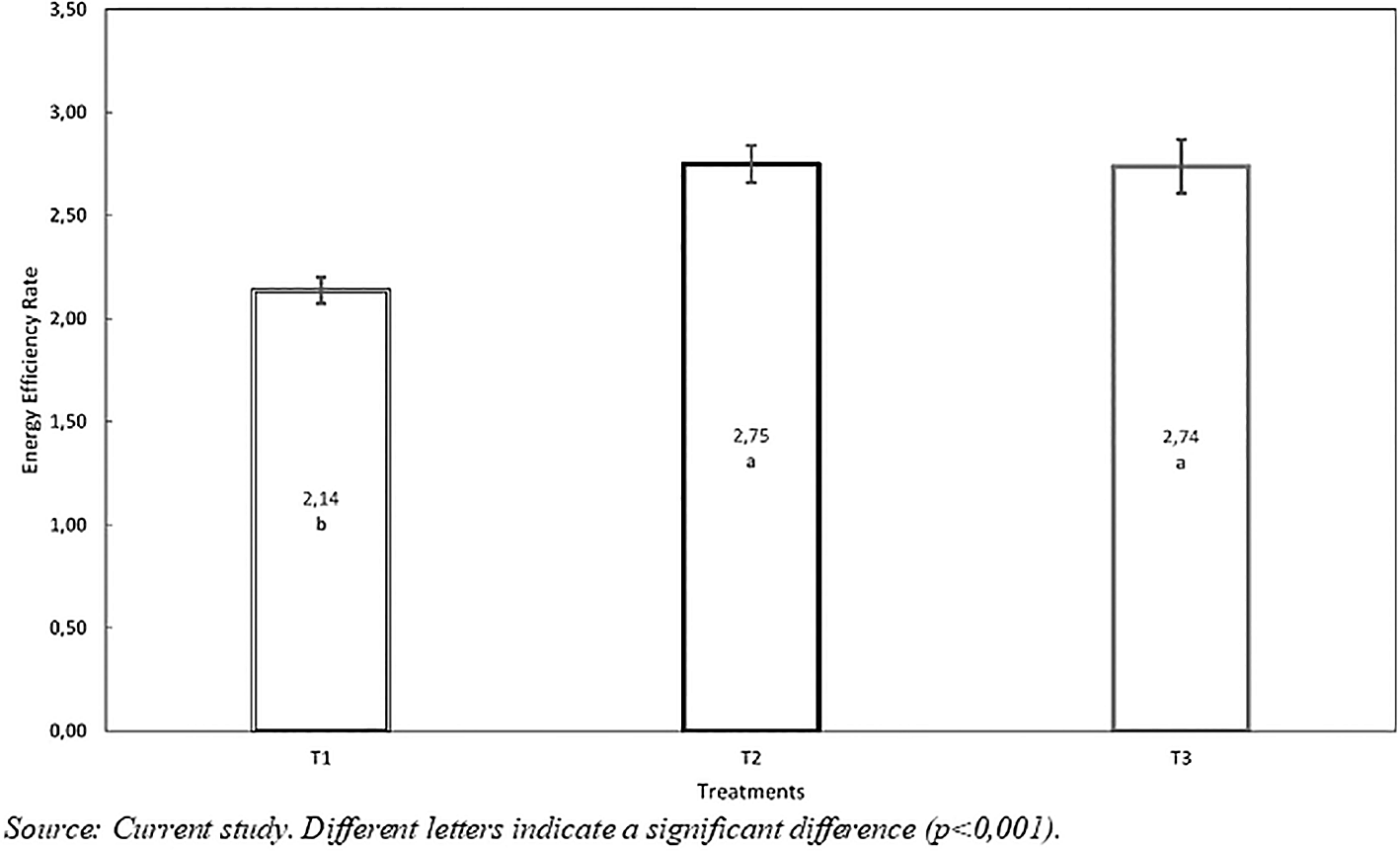
Energy efficiency ratio with hydrolyzed red worm ( *Eisenia foétida*).

The T1 and T2 treatments showed significant differences (p<0.001) compared to the control diet T1, which was surpassed by T2 and T3 by 0.61 and 0.60 kcal/g, respectively (see Figure 5). This indicates better utilization and performance in the metabolic energy of lipids and proteins. The differences obtained are determined by the different inclusion levels of the hydrolyzed in the diets. This could be due to the quantity and nutritional quality of the hydrolyzed used, demonstrating once again that appropriate inclusion levels can constitute a viable protein and energy source in red tilapia ( *Oreochromis sp.*) production, reiterating the efficient use of the nutrients present in the hydrolyzed. ^12, 32, 34^

Figure 5 shows that treatments T2 and T3 achieved better energy efficiency ratio (2.75 and 2.74) compared to T1 (2.14) without hydrolyzed (p<0.001).

Similarly, the greater availability of energy could be related to the acidification of the diet generated by the hydrolyzed, which facilitates nutrient digestibility and, consequently, fish growth. ^32^ Additionally, endogenous proteases may have influenced protein solubility, favoring energy absorption and utilization in the fish. The results obtained in this study for the Energy Efficiency Ratio (EER) are superior to those reported by Martinez-Castillo et al., ^31^ who evaluated the zootechnical performance of *Piaractus brachypomus* (cachama) fry fed with different levels of gross energy, obtaining results between 0.35 and 0.41%. They are also superior to those reported by Botello-Leon et al., ^35^ who evaluated the substitution of fishmeal with protein cane meal for fattening *Oreochromis sp.* (red tilapia), finding that the diets presented significant conversion rates with an appropriate protein rate-energy ratio of 0.94 and 0.96 of digestible energy values.

Diet cost

It is important to note that with a higher inclusion of hydrolyzed, the cost of the diet decreases significantly, showing statistical differences (p<0.05) between treatments with and without hydrolyzed inclusion ( Table 6). T3 (20%) presented the lowest cost, followed by T2 and T1. Regarding this, there is a difference between the price of the control diet (T1) and T3 of $0.05 per kg, and with T2 of $0.04 per kg, representing a cost reduction of 5.8% and 4.6%, respectively.

Similar behavior has been reported in studies with fish hydrolyzed in red tilapia feed, ^36^ rainbow trout, ^37^ and even broilers, ^38^ were increasing the inclusion of silage in the diet significantly decreases feed cost.

The results of this study are similar to those reported by Llanes y Parisi, ^32^ where they replaced fishmeal with chemically made silages using sulfuric and formic acids with fish by-products in extruded diets for *Clarias gariepinus.* They also align with those cited by Vilchez, ^39^ who evaluated three inclusion levels of poultry by-product meal in finishing diets for *Piaractus brachypomus*, where higher inclusion levels in the evaluated diets significantly reduced the price per kilogram of feed. Similarly, they match the findings of Perea et al., ^19^ who conducted an economic evaluation of the use of fish waste silage in the feed of *Oreochromis sp.*

## Conclusions

Hydrolyzed California red worm ( *Eisenia foétida*) is a high-quality nutritional alternative for use in diets for red tilapia at inclusion levels of 10% and 20%, optimizing protein and energy efficiency, weight gain, and feed conversion rate. In addition, the inclusion of the hydrolyzed could provide an interesting economic profit due to the lower cost of the diet for small and medium-scale fish producers.

## Ethics statement

Animal Welfare Protocol in the study “Effect of hydrolyzed red worm (Eisenia foétida) on production parameters in red tilapia (Oreochromis sp.)”

At the beginning of the document, you can see the approval: The Ethics Committee for Scientific Research of the University of Cauca, endorses the project according to minutes No. 6.1-1.25/15 of August 5, 2019. ^40^

Animal ethics: Every effort was made to mitigate the suffering of the animals. Procedures were also implemented in strict compliance with animal welfare standards. All necessary measures were taken to recreate the natural habitat conditions of the species, in order to minimise pain, stress and suffering of the animals, ensuring their well-being at all times. The measures and conditions adopted are detailed in the Declaration of Commitment to Animal Welfare https://doi.org/10.5281/zenodo.14477924 ^41^

## Data Availability

The project contains the following underlying data: Statistics “Effect of hydrolyzed red worm (Eisenia foétida) on production parameters in red tilapia (Oreochromis sp.)”.XLSX [Data set]. Zenodo. https://doi.org/10.5281/zenodo.14081025 ^42^ Data are available under the terms of the Creative Commons Attribution 4.0 International license (CC-BY 4.0). Arrive 2.0: ARRIVE checklist for ‘Effect of hydrolyzed red worm (Eisenia foétida) on production parameters in red tilapia (Oreochromis sp.) ‘ https://doi.org/10.5281/zenodo.13139560 ^43^ Data are available under the terms of the Creative Commons Attribution 4.0 International license (CC-BY 4.0).
