# Supplementary material for: Effect of hydrolyzed red worm ( Eisenia foétida) on production parameters in red tilapia ( Oreochromis sp.)
Source: F1000Res. 2025 Jan 15;14:90. [Version 1] doi: 10.12688/f1000research.154622.1 (PMC11916745; doi:10.12688/f1000research.154622.1)
Supplement: Peer review history

# Reviewer report: Reviewer response for version 1

**DOI:** 10.5256/f1000research.169671.r368929

**Published:** 2025-03-14

**Authors:** Zulhisyam Abdul Kari

**Affiliations:** 1 Universiti Malaysia Kelantan, Kafrelsheikh University, Universiti Sains Malaysia, Kelantan, Malaysia

Here are some **minor comments** for the article:

**General Comments**

1. **Clarity in Introduction:**
  - The introduction is well-structured, but it would benefit from a clearer problem statement. The rationale for using hydrolyzed red worm (HRW) should be explicitly connected to existing challenges in aquaculture feed.
  - Consider incorporating more references on the use of insect or worm-based protein sources in aquaculture to strengthen the background.
  - Please also read the latest article published which can help the authors in the introduction part as below: (reference 1 to 9)

My intention was not to self-promote but rather to provide the authors with relevant and recent references that could enhance their study, particularly in the context of aquaculture nutrition. The suggested citations serve as a reference point for the authors to:

1. **Improve the Introduction Section** – The cited works illustrate how introductions in similar aquaculture nutrition studies are structured, integrating the latest research trends and background information in a comprehensive manner. This guidance aims to help the authors refine their context-setting and literature synthesis.
2. **Ensure the Use of Up-to-Date References** – The field of aquaculture nutrition is rapidly evolving, and my recent work contains key insights that are directly relevant to the study’s focus on red tilapia and alternative protein sources. Citing recent studies, including my own, ensures that the manuscript aligns with the latest scientific developments.
3. **Enhance Scientific Rigor** – By referring to well-documented findings in related studies, the authors can strengthen their discussion, reinforce their hypotheses, and make well-supported conclusions. My recommendations were purely scientific, aimed at improving the quality of the manuscript rather than for personal or professional gain.

1. **Methodology Enhancements:**
  - The justification for selecting 0%, 10%, and 20% HRW inclusion levels should be elaborated. Were these levels based on prior studies, preliminary trials, or nutrient availability considerations?
  - In the water quality monitoring section, specifying how often parameters were measured (daily, weekly) would enhance reproducibility.
  - The statistical analysis section should clarify whether assumptions (e.g., normality and homogeneity of variance) were tested before applying ANOVA.
2. **Results Interpretation:**
  - The phrase "no statistical difference (p<0.217)" should be revised to "p > 0.05, indicating no significant difference" for clarity.
  - While weight gain showed significant improvement at 10% inclusion, size increase did not. The discussion should explicitly state whether size growth is a limiting factor or if hydrolyzed proteins primarily contribute to mass gain.
  - Since no significant difference was found between 10% and 20% HRW inclusion, a cost-benefit analysis might help determine whether 10% is the optimal level.
3. **Discussion and Conclusion Adjustments:**
  - The conclusion should specify that while weight gain and feed efficiency improved, size growth did not show a significant difference across treatments.
  - Highlighting potential long-term effects (e.g., immune response, fillet quality, or digestive enzyme activity) could add depth to future research recommendations.
  - The limitations of the study should be acknowledged, such as the relatively short experimental duration (30 days) and the need for trials at different life stages.

**Minor Corrections:**

1. Spelling & Formatting
  - *Eisenia foétida* → should be consistently italicized as *Eisenia foetida* throughout the text.
  - "size increasse" (Figure 2 caption) → "size increase"
  - "feed conversión" (Figure 3 caption) → "feed conversion"
2. Figures & Tables:
  - Ensure all figures have clear legends and units where applicable (e.g., Figure 5 on Energy Efficiency Ratio).
  - The proximate composition of HRW could be compared more explicitly with fish meal in a supplementary table.

By implementing these minor suggestions, the article can improve clarity, precision, and scientific rigor while maintaining its strong contribution to aquaculture research.

Is the work clearly and accurately presented and does it cite the current literature?

Yes

If applicable, is the statistical analysis and its interpretation appropriate?

Yes

Are all the source data underlying the results available to ensure full reproducibility?

Yes

Is the study design appropriate and is the work technically sound?

Partly

Are the conclusions drawn adequately supported by the results?

Partly

Are sufficient details of methods and analysis provided to allow replication by others?

Yes

Reviewer Expertise:

Aquaculture nutrition

I confirm that I have read this submission and believe that I have an appropriate level of expertise to confirm that it is of an acceptable scientific standard.

## References

[1] : The possibility of replacing fish meal with fermented soy pulp on the growth performance, blood biochemistry, liver, and intestinal morphology of African catfish (Clarias gariepinus). *Aquaculture Reports* .2021;21: 10.1016/j.aqrep.2021.100815 10.1016/j.aqrep.2021.100815

[2] : Exploring the Roles of Dietary Herbal Essential Oils in Aquaculture: A Review. *Animals (Basel)* .2022;12(7) : 10.3390/ani12070823 10.3390/ani12070823 35405814 PMC8996993

[3] : Dose response effects of dietary clove and peppermint oils on the growth performance, physio-metabolic response, feed utilization, immunity, and organ histology in African catfish (clarias gariepinus). *Vet Res Commun* .2025;49(2) : 10.1007/s11259-025-10660-9 101 10.1007/s11259-025-10660-9 39920512

[4] : The potential of hydrolyzed chicken feather meal as a partial replacement for fish meal and its effects on the growth and health status of African catfish (Clarias gariepinus) fingerlings. *Aquaculture International* .2025;33(3) : 10.1007/s10499-025-01847-7 10.1007/s10499-025-01847-7

[5] : Effects of dietary Spirulina platensis supplementation on growth performance, whole body composition, antioxidant activity, histological alterations, and resistance to Vibrio parahaemolyticus in Pacific white shrimp, Litopenaeus vannamei. *Aquaculture Reports* .2025;40: 10.1016/j.aqrep.2024.102606 10.1016/j.aqrep.2024.102606

[6] : Effects of nano-selenium and/or vitamin E supplementation on growth performance, antioxidant status, histopathology and resistance to Aspergillus flavus in Nile tilapia (Oreochromis niloticus). *BMC Vet Res* .2025;21(1) : 10.1186/s12917-025-04471-y 50 10.1186/s12917-025-04471-y 39910528 PMC11800638

[7] : Correction to: The effects of nano‑selenium and/or vitamin C on the growth performance, blood health, organ histology, molecular alterations, and disease resistance of Nile tilapia (Oreochromis niloticus) against Saprolegnia ferax. *Aquaculture International* .2025;33(1) : 10.1007/s10499-024-01783-y 10.1007/s10499-024-01783-y

[8] : Nutrient Profiling of Some Terrestrial Plant Leaves for Their Prospective Use as Nonconventional Ingredients in Carp Diets. *Aquaculture Research* .2025;2025(1) : 10.1155/are/9989338 10.1155/are/9989338

[9] : Exploring the role of plant oils in aquaculture practices: an overview. *Aquaculture International* .2024;32(6) : 10.1007/s10499-024-01538-9 7719-7745 10.1007/s10499-024-01538-9

# Reviewer report: Reviewer response for version 1

**DOI:** 10.5256/f1000research.169671.r370242

**Published:** 2025-03-11

**Authors:** Fredrick Syanya

**Affiliations:** 1 Cochin University of Science and Technology, Kochi, India

The research is interesting since it's using red worms (Eisenia foetida) with a CP of 62% as a source of protein, which can be a perfect replacement for fish meal that has been traditionally used as the main source of protein for fish feed formulation.

 However, Expound on your introduction, as extensive work has been done in this area by incorporating more literature.

 Let your problem statement come out clearly in the introduction.

 The methodology is sound; however, it could be improved on some parameters.

 What informed the diet percentage determination? Elaborate in your work. 0% hydrolysed worm, 10% hydrolysed worm, and 20% hydrolysed worm.

 Instead of presenting no statistical differences (p<0.217), you would rather say p>0.05 for a 95% chance of occurrence (page 8).

 Elaborate more in your conclusion and give clear recommendations on the percentage dosage that is appropriate for red tilapia fingerlings.

 Highlight the limitations of this study.

Is the work clearly and accurately presented and does it cite the current literature?

Yes

If applicable, is the statistical analysis and its interpretation appropriate?

Partly

Are all the source data underlying the results available to ensure full reproducibility?

Yes

Is the study design appropriate and is the work technically sound?

Yes

Are the conclusions drawn adequately supported by the results?

Partly

Are sufficient details of methods and analysis provided to allow replication by others?

Yes

Reviewer Expertise:

Aquaculture

I confirm that I have read this submission and believe that I have an appropriate level of expertise to confirm that it is of an acceptable scientific standard, however I have significant reservations, as outlined above.

# Reviewer report: Reviewer response for version 1

**DOI:** 10.5256/f1000research.169671.r363739

**Published:** 2025-02-14

**Authors:** Farah Izana Abdullah

**Affiliations:** 1 International Institute of Aquaculture and Aquatic Sciences, Universiti Putra Malaysia, Port Dickson, Malaysia

**1. Summary of the Article**

 The study investigates the use of hydrolyzed red worm (HRW) as a protein source in red tilapia ( *Oreochromis sp.*) diets. The experiment involved 90 red tilapia fingerlings distributed across nine aquariums, with three dietary treatments:

- **T1 (Control Diet, 0% HRW)**
- **T2 (10% HRW inclusion)**
- **T3 (20% HRW inclusion)**

The key parameters evaluated included weight gain, size increase, feed conversion ratio (FCR), protein efficiency rate (PER), energy efficiency ratio (EER), and diet cost.

**Key Findings:**

- **Weight gain:** T2 (10%) showed significantly higher weight gain compared to T1 (p=0.047). No significant difference was observed between T2 and T3.
- **Size increase:** No statistically significant difference was found among treatments (p=0.217).
- **FCR, PER, and EER:** Significant improvements were observed in T2 and T3 compared to T1 (p=0.001).
- **Diet cost:** Higher HRW inclusion reduced the overall feed cost (p=0.034).
- **No mortality observed:** Water quality remained within acceptable limits.

The authors conclude that HRW is a viable and cost-effective protein source for red tilapia.

**2. Assessment of Data Availability and Reproducibility**

**Are all the source data underlying the results available to ensure full reproducibility?**

**Yes, but verification is needed.**

- The authors provide access to a dataset hosted on Zenodo ( **DOI: 10.5281/zenodo.14081025**).
- The article states compliance with ARRIVE 2.0 guidelines, ensuring transparency in animal research.

**Recommendations for Improvement:**

- Verify that the dataset contains all raw data, including:
  - Individual fish measurements (initial and final weight, size)
  - Daily feed intake per tank
  - Water quality parameters over time
  - Statistical analysis scripts (if applicable)
- Ensure metadata is provided for full reproducibility.

**3. Evaluation of the Scientific Soundness of the Conclusions**

**Are the conclusions drawn adequately supported by the results?**

**Partly.** The study presents strong evidence supporting the benefits of HRW inclusion, but certain aspects require further clarification or additional data to strengthen the conclusions.

**Strengths:**

 ✔ **Statistically significant improvements in key parameters:** The weight gain, FCR, PER, and EER results support the claim that HRW enhances growth and feed efficiency. ✔ **Economic benefit:** The reduced diet cost further justifies HRW as an alternative protein source. ✔ **No negative health effects:** The lack of mortality and stable water quality parameters indicate that HRW does not introduce harmful effects on tilapia health.

**Weaknesses and Required Revisions:**

**1. Lack of Statistically Significant Improvement in Size Increase**

- The study finds no significant improvement in size increase (p=0.217), yet the conclusion broadly states HRW "improves production parameters."
- **Required Revision:** Clearly specify that while weight gain improved, length growth did not show a statistically significant difference.

**2. Lack of Clarity on Optimal Inclusion Level**

- No significant difference was observed between **T2 (10%) and T3 (20%)** in growth performance, yet both are recommended.
- **Required Revision:** The authors should discuss whether 10% is the optimal level since increasing to 20% did not yield additional benefits.
- If possible, a cost-benefit analysis should be included to determine the most economically viable HRW level.

**3. Short Experimental Duration (30 Days)**

- Long-term effects on fish growth, health, or reproductive performance were not evaluated.
- **Recommended Improvement:** Acknowledge this limitation and suggest future research on extended feeding trials.

**4. Need for More Discussion on Protein and Energy Utilization**

- The article attributes improved feed efficiency to better protein digestibility but does not detail the amino acid profile of HRW.
- **Recommended Improvement:** If amino acid analysis is available, include a discussion on how HRW’s amino acid profile compares to conventional fish meal.

**4. Final Recommendations**

 To improve the scientific rigor of the paper and ensure clarity in conclusions, the following revisions are **mandatory** for scientific soundness:

1. **Clarify that size increase was not significantly improved** and discuss possible reasons.
2. **Reassess the recommendation of 20% HRW inclusion**, given that 10% yielded similar results.
3. **Acknowledge the short duration of the study** and the need for long-term assessments.
4. **Ensure the dataset is fully detailed and contains all necessary raw data** for reproducibility.

Additionally, the following improvements are **strongly recommended** to enhance clarity:

- Provide more discussion on **HRW’s amino acid composition**.
- If possible, conduct a **cost-benefit analysis** to determine the most practical HRW inclusion level.

Is the work clearly and accurately presented and does it cite the current literature?

Partly

If applicable, is the statistical analysis and its interpretation appropriate?

Yes

Are all the source data underlying the results available to ensure full reproducibility?

Yes

Is the study design appropriate and is the work technically sound?

Yes

Are the conclusions drawn adequately supported by the results?

Partly

Are sufficient details of methods and analysis provided to allow replication by others?

Yes

Reviewer Expertise:

My expertise includes protein hydrolysate, particularly its nutritional characteristics, bioactive properties, extraction methods, and applications in aquaculture, functional foods, and pharmaceuticals.

I confirm that I have read this submission and believe that I have an appropriate level of expertise to confirm that it is of an acceptable scientific standard, however I have significant reservations, as outlined above.
